# An adaptive brain actuated system for augmenting rehabilitation

**DOI:** 10.3389/fnins.2014.00415

**Published:** 2014-12-12

**Authors:** Scott A. Roset, Katie Gant, Abhishek Prasad, Justin C. Sanchez

**Affiliations:** ^1^Department of Biomedical Engineering, University of MiamiCoral Gables, FL, USA; ^2^Miami Project to Cure Paralysis, University of MiamiCoral Gables, FL, USA

**Keywords:** brain-computer interface, error-related potentials, reinforcement learning, neurorehabilitation, spinal cord injuries

## Abstract

For people living with paralysis, restoration of hand function remains the top priority because it leads to independence and improvement in quality of life. In approaches to restore hand and arm function, a goal is to better engage voluntary control and counteract maladaptive brain reorganization that results from non-use. Standard rehabilitation augmented with developments from the study of brain-computer interfaces could provide a combined therapy approach for motor cortex rehabilitation and to alleviate motor impairments. In this paper, an adaptive brain-computer interface system intended for application to control a functional electrical stimulation (FES) device is developed as an experimental test bed for augmenting rehabilitation with a brain-computer interface. The system's performance is improved throughout rehabilitation by passive user feedback and reinforcement learning. By continuously adapting to the user's brain activity, similar adaptive systems could be used to support clinical brain-computer interface neurorehabilitation over multiple days.

## Introduction

Motor impairment may occur due to spinal cord injury (SCI), traumatic brain injury (TBI), stroke, and other neurodegenerative disorders and diseases. For people living with paralysis, restoration of hand function remains the top priority because it leads to independence and improvement in quality of life (Anderson, 2004; Wyndaele and Wyndaele, 2006). In approaches to restore hand and arm function, a goal is to better engage voluntary control and counteract maladaptive brain reorganization that results from non-use (Hoffman and Field-Fote, 2006). For these reasons, there is a need for new therapies that will help restore motor abilities and rehabilitate the motor cortex. Standard rehabilitation augmented with developments from the study of Brain Computer Interfaces (BCI) could provide a combined therapy approach (Daly and Wolpaw, 2008; Daly et al., 2009) for motor cortex rehabilitation and to alleviate motor impairments. The appeal of combined approaches to rehabilitation is that they can engage top-down control from the central nervous system (CNS) and couple it with bottom-up peripheral therapy. A BCI can bypass the injury and connect the brain to peripheral muscles via functional stimulation. To implement this approach, BCIs record a user's brain activity by electroencephalography (EEG) and translate it into actions for the user to control (Wolpaw et al., 1991; Pfurtscheller et al., 2000, 2003; Galán et al., 2008; Do et al., 2011; McFarland and Wolpaw, 2011; LaFleur et al., 2013). Combining BCIs with activation of paralyzed muscle by electrical stimulation is one example of how a combined therapy could provide a unique approach to motor cortex rehabilitation by enabling the user to actively control extremities during rehabilitation with brain activity (Daly and Wolpaw, 2008).

Several groups have recently developed BCIs combined with rehabilitation to produce new combined therapies (Daly et al., 2009; Ang et al., 2010; Broetz et al., 2010; Várkuti et al., 2013). However, one key challenge in these approaches is the changes in the motor cortex that occurs during rehabilitation over long durations (Cramer et al., 2007). Since the motor cortex is the primary input to the BCI, changes in the motor cortex could affect the BCI's performance. In traditional BCI approaches, the system must be recalibrated at the beginning of each session to initialize to high performance. This approach is not well suited to continuous BCI-rehabilitation use over long durations spanning from days to years. An alternative to this approach is to maintain the system's performance throughout rehabilitation with an adaptive BCI. Several approaches have used an initial training set with supervised learning to calibrate the system and unsupervised learning to adapt the system online (Li and Guan, 2008; Li et al., 2008; Vidaurre et al., 2011). These approaches have a limited ability to adapt since the adaption relies on the supervised learning from the training set.

We have developed adaptive decoders based on reinforcement learning (RL) that could maintain an adaptive BCI's performance throughout rehabilitation because the RL BCI continuously adapts to the user (Mahmoudi and Sanchez, 2011). The system adapts the BCI's mapping of brain activity to action when the user perceives the action was incorrect and generates an error-related potential (ErrP) (Ferrez and Millan, 2008). Similarly, the system reinforces the mapping of brain activity to action when the user perceives the action was correct. Given ErrPs will be more stable over time than motor potentials, building a ErrP classifier and then using it to train a motor potential classifier is better than building a motor potential classifier from a training set (Cramer et al., 2007; Ferrez and Millan, 2008). The system developed in this study is unique compared to existing systems as it does not require calibration prior to each session.

In this paper, a new EEG BCI system using reinforcement learning intended for application to control a functional electrical stimulation (FES) device is developed as an experimental test bed for augmenting rehabilitation with a BCI. We validate the RL learning architecture developed to use with the BCI. We compare the decoder characteristics in a closed-loop environment between an uninjured control and a subject living with SCI. Our results indicate the BCI could continuously adapt to both the control and SCI subject and the performance improved over 4 sessions spanning 1 week of use and without daily initialization.

## Methods

### Study participants

The system design function was demonstrated and compared between a control subject and a subject with a chronic SCI. All procedures followed in the study were approved by the University of Miami Institutional Review Board. The subjects provided written informed consent. The inclusion criteria followed for recruiting subjects with SCI included: chronic injury (longer than 1 year), no denervation of target muscles, and C5 or C6-level motor complete injury classified by the American Spinal Injury Association (ASIA) standards (Marino et al., 2003). Both subjects were 30 years old males. The subject with SCI was injured playing football, and his injury (duration = 15 years) was classified by ASIA standards as incomplete (ASIA B), with bilateral motor levels of C6. Motor scores of 5 (normal function) were attained at the C5-level bilaterally, with scores of 5 (right) and 3 (left) at the C6-level. All motor scores below level C6 were zero. The subjects had no history of other serious medical issues.

### Experimental task

Hand grasp/open function was chosen as the experimental task as restoration of hand/arm function is the highest priority for people with tetraplegia (Anderson, 2004). The goal of the task was to enable direct brain actuation of hand closing and opening. In addition to extracting motor potentials, an evoked potential from the brain was of interest: error potentials (ErrP) which are generated when an error is observed. In this experiment, ErrPs were generated when the user perceived the action of the BCI was incorrect. Both motor and error potentials are necessary for conducting closed-loop reinforcement learning in this context.

**Figure 1:**
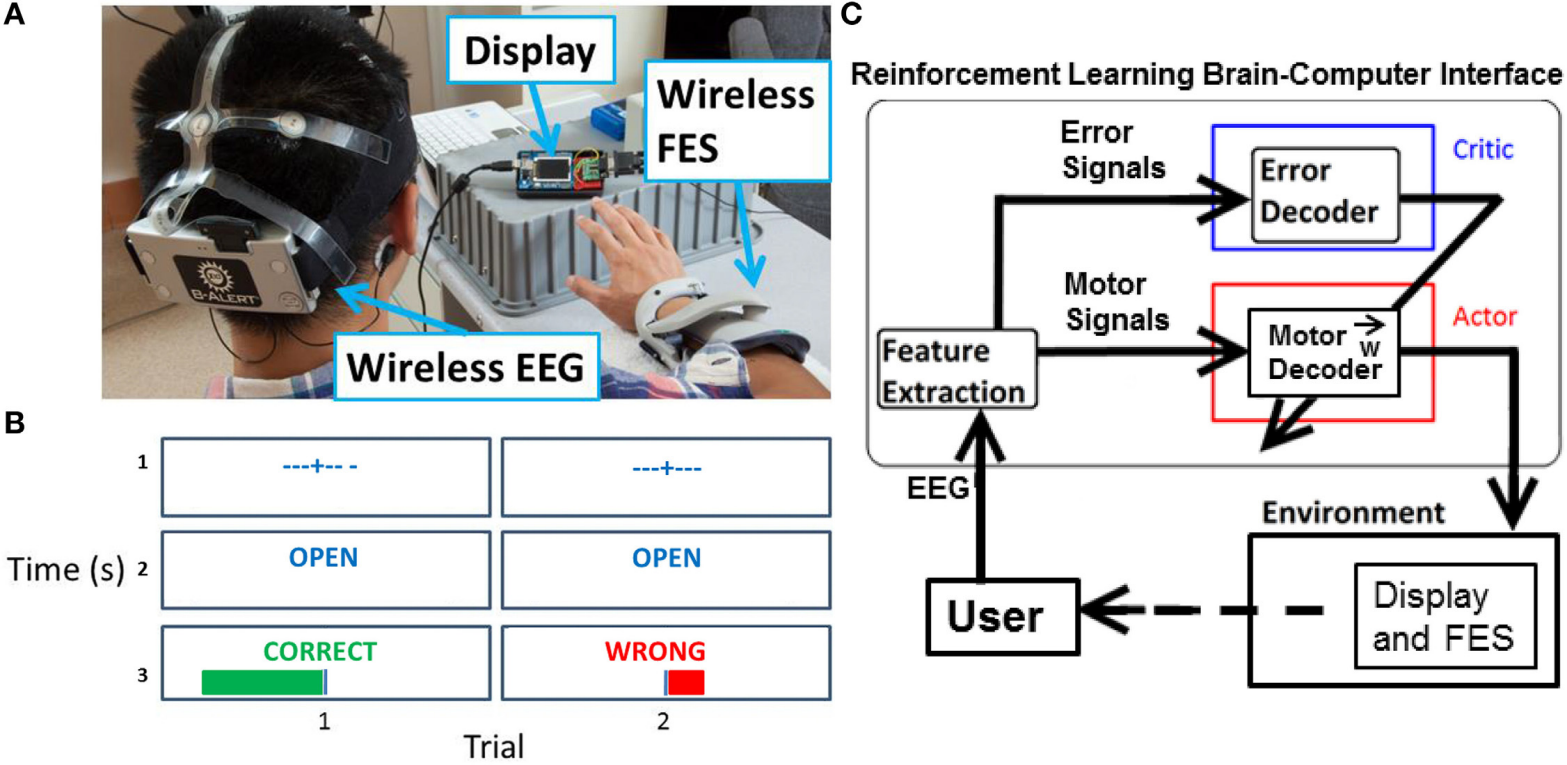
**Experiment setup overview. (A)** Visible are the EEG headset, display, and FES. **(B)** For each trial during the experimental task, the display showed a fixation cross, followed by a cue for “open” or “close” for 1 s, and then feedback of “correct” or “wrong” for 1 s. A magnitude plot also showed the unthresholded output of the motor potentials decoder. **(C)** Actor-critic RL BCI architecture. The actor decodes motor potentials and outputs an action. The critic detects an ErrP and provides feedback to actor. The actor uses feedback from the critic to adapt to the user.

A preliminary session was used to collect representative ErrPs to develop an ErrP classifier. During the preliminary session, feedback was random and approximately 50% of the 120 trials resulted in a “wrong” outcome. No stimulation was delivered to the subjects during the preliminary session. The subjects sat facing a display with their right forearm resting on a table (Figure 1A). After a fixation cross was shown on the display for 3 s to minimize eye movements, cues of “open” or “close” were presented for 1 s that instructed the person to either open or close his hand. Random visual feedback of “correct” or “wrong” was then shown for 1 s, along with a corresponding plot of the unthresholded output of the system (Figure 1B, row 3).

Four closed-loop sessions were performed and consisted of 300 trials during the 1st session, 450 trials each during the 2nd and 3rd sessions, and 300 trials during the 4th session. Time between sessions was varied to test the adaptation of the network with 2 days between the 1st and 2nd sessions, 4 days between the 2nd and 3rd sessions, and 1 day between the 3rd and 4th sessions. During closed-looped sessions, ErrPs were collected and used to adapt the BCI. The same visual cues were displayed on the screen as in the preliminary session. However, in closed-loop sessions the displayed feedback matched the output of the adaptive BCI. When the output of the adaptive BCI was determined to be “open,” FES was delivered to the hand muscles. No FES was delivered for trials when the output of the adaptive BCI was “close”. All trials were used in the analysis.

### Neural data acquisition

A wireless 9-channel EEG system (256 Hz sampling rate, 16-bits of resolution, X10 headset, Advanced Brain Monitoring, Carlsbad, CA) was fitted to the subject's head (Figure 1A). Electrodes (Fz, F3, F4, Cz, C3, C4, POz, P3, P4) were arranged according to the International 10–20 system standards. Foam sensors attached to the sensor sites on the headstrips were saturated with Synapse (Kustomer Kinetics, Arcadia, CA) conductive electrode paste and the corresponding sites on the head were abraded and cleaned before placing the sensors on the scalp. Electrode impedances were tested before and after each experimental session using the manufacturer provided software.

To differentiate between brain activity associated with motor potentials and ErrPs a different channel of EEG was used for each potential. Motor potentials for the intent to open or close the hand were recorded from the C3 electrode, and ErrPs were recorded from the Cz electrode. Frequencies of 1–50 Hz were used for the motor potential decoder, and frequencies of 1–12 Hz were used for the ErrP decoder (Qin et al., 2004; Ferrez and Millan, 2008). For motor potentials, EEG generated between 0.15 and 1.0 s after the display of cues (“open” or “close”) was used. For ErrPs, EEG generated from 0.15 to 0.70 s after display of feedback (“correct” or “wrong”) was used. EEG was transformed into the frequency domain using the Fast Fourier Transform (FFT) to obtain a power spectral density (PSD) of 1 Hz resolution. The input features to both decoders were normalized PSD z-scores (LeCun et al., 1998), which were found by subtracting the mean of all previous trials at each frequency and dividing by the standard deviation of all previous trials for that frequency. A negative z-score means for that trial and frequency the power was below the mean.

### Muscle stimulation

A neuroprosthetic wrist-hand orthosis (NESS H200, Bioness Inc., Valencia, CA) was fitted to the right hand of the subject. FES was delivered to the extensor muscles (extensor digitorum communis and extensor pollicis brevis) to produce opening movements of the fingers and hand. Stimulation intensity was set by holding the pulse duration (300 μs) and frequency (35 Hz) constant, while slowly increasing the current amplitude. Once a maximal muscle contraction was attained (i.e., increases in current intensity did not produce additional muscle contraction), the current amplitude was increased an additional 25% in order to maintain consistent muscle contractions throughout the experiment. No stimulus artifact was observed in subsequent recordings.

### Actor-critic reinforcement learning architecture

The adaptive BCI is based on an actor-critic RL architecture (Figure 1C) (Mahmoudi and Sanchez, 2011). The actor decodes motor potentials from the user to determine the user's intent to open or close the hand. The critic provides feedback to the actor by detecting ErrPs generated by the user (Falkenstein et al., 2000). The actor-critic RL algorithm is a semi-supervised machine learning algorithm that optimizes the actor's decoding of the user's motor potentials based on feedback from the critic (Sutton and Barto, 1998).

The actor is parameterized by a 3-layer fully connected feedforward neural network. The hidden and output nodes of the neural network perform a weighted sum on their inputs. The weighted sum at each node is passed through a hyperbolic tangent function with an output in the range of −1 to 1. The weights between the actor's nodes are initialized randomly and then updated after each trial based on feedback. The actor's weights update can be expressed as:

(1) $$\Delta w_{ij}=\gamma f(x_{i}(p_{j}-x_{j}))+\gamma (1-f)(x_{i}(1-p_{j}-x_{j}))$$

Here *w_ij_* is the weight connecting nodes *i* and *j*, γ is the learning rate, *p_j_* is a sign function of output *x_j_* (positive values become +1 and negative values become −1) and f is feedback from the critic. The weight update equation is based on Hebbian style learning (Mahmoudi and Sanchez, 2011; Pohlmeyer et al., 2012). The critic provides the feedback by decoding the user's EEG to determine if an ErrP was generated. If an ErrP is detected, a feedback of −1 is provided to the network for adaptation. If not, a feedback value of 1 is given. The functional mapping between neural activity and behavior in the actor is constructed using the weight update equation (Equation 1).

### Adaptive BCI usage

**Figure 2:**
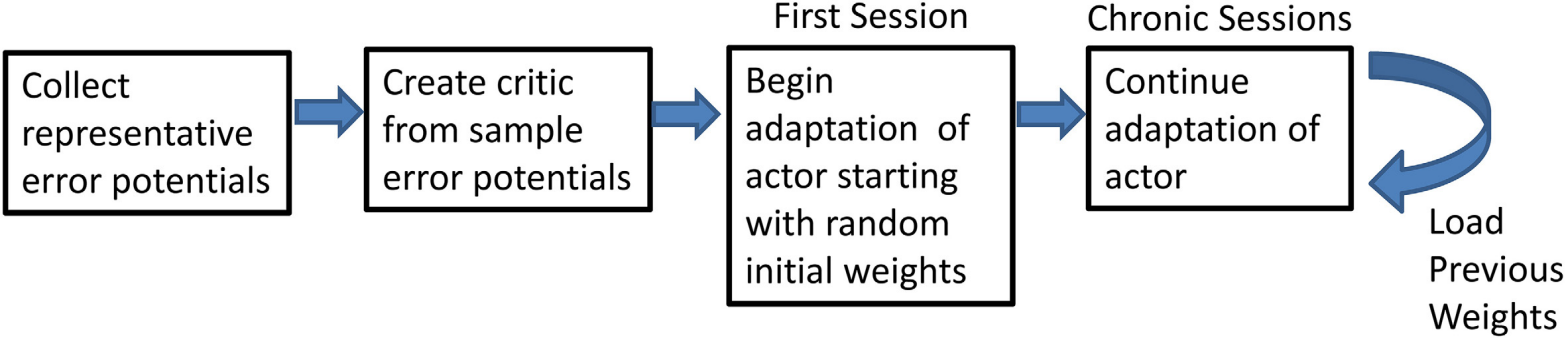
**Flowchart of the steps of the experiment**. Flowchart shows the preliminary steps of the experiment and how the final step can be repeated.

Adaptive BCI usage was broken down into several intermediate steps (Figure 2). Representative ErrPs were collected in the preliminary session and used to develop the critic through supervised learning (Prechelt, 1998). Once the critic was created, the weights of the actor were initialized to random initial values and trained through RL and feedback from the critic. After the first closed-loop session, in which the weights are initialized to random values, all subsequent closed-loop sessions used the weights from the previous session with no offline adjustments.

### Critic as error potential classifier

The error potential classifier “critic” detects ErrPs in the user's EEG to determine if the user perceived that an error occurred. The critic then provides binary feedback, −1 or 1, to the actor. The input to the error potential classifier was the normalized PSD from 1–12 Hz in 1 Hz bins computed on the 0.15–0.70 s of EEG data after the actor's output (action) was shown on the display.

The error potential classifier in the critic is a 3-layer neural network with 12 inputs nodes, for the 1–12 Hz in 1 Hz bins, and 5 hidden nodes. Representative ErrPs were collected in the 120 trials of the preliminary session and were randomly assigned to a training set or test set, approximately 60 trials each. The training set was used to optimize the neural network's weights of the critic with supervised learning. The weights produced from different initial seeds were assessed by applying them to the test set and computing the classification accuracy. The weights with the best classification accuracy were used for closed-loop sessions.

To test the critic training procedure during the preliminary data collection, 10-fold cross validation was performed (Table 1). The minimum and maximum accuracy in the 10-fold cross validation were within 5% of the mean accuracy, showing that the critic should have reasonable performance during the closed-loop sessions.

**Table 1:** **Classification results of the critic**.

	**SCI**			**Control**	
	**Predicted correct**	**Predicted error**		**Predicted correct**	**Predicted error**
Correct	66.8 ± 4.1	33.2 ± 4.1	Correct	68.6 ± 2.6	31.4 ± 2.6
Error	40.8 ± 5.0	59.2 ± 5.0	Error	30.9 ± 3.6	69.1 ± 3.6

*Ten-fold cross validation results of the critic for both the control and SCI subject*.

## Results

### Closed-loop trials

**Figure 3:**
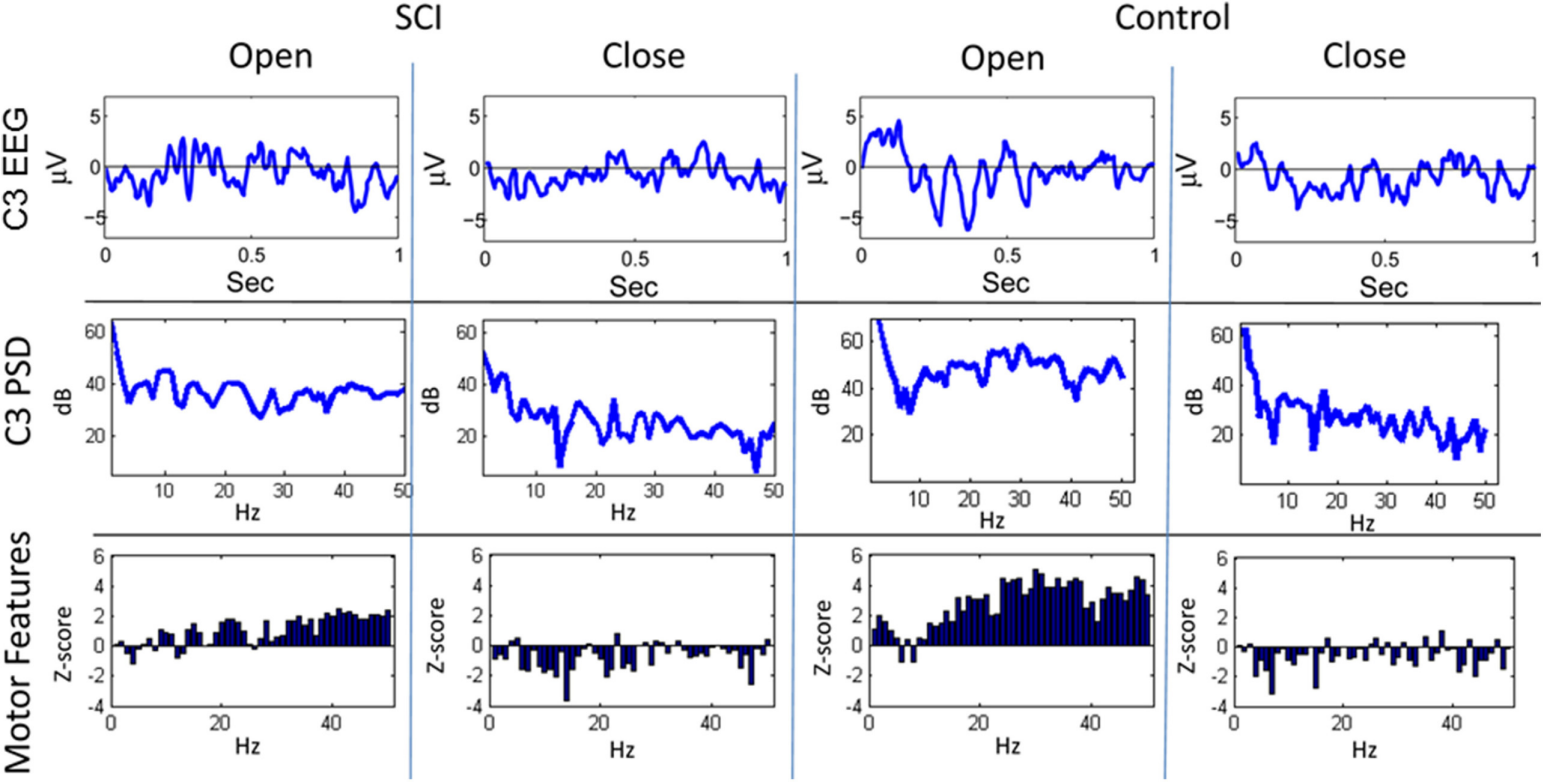
**Sample trials from closed-loop sessions for cues**. Columns show samples for cues of “open” and “close” for both the SCI and control subject. Rows show raw EEG from electrode C3, PSD, and features.

**Figure 4:**
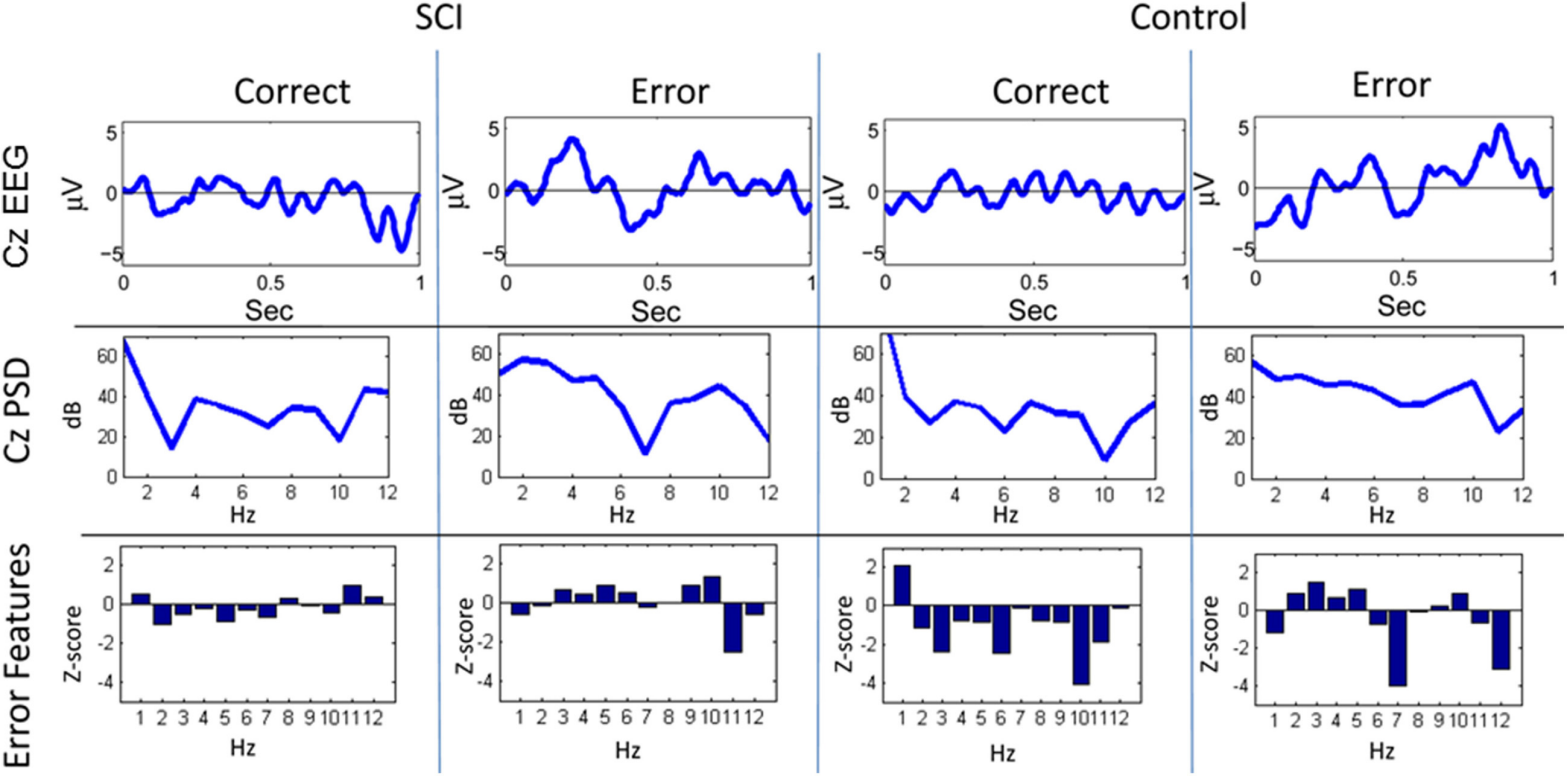
**Sample trials from closed-loop sessions for feedback**. Columns show samples for feedback of “correct” and “error” for both the SCI and control subject. Rows show raw EEG from electrode Cz, PSD, and features.

Figures 3, 4 show representative trials from the closed-loop experiments and give insight into how the system processes the EEG to create features for the classifiers. The first row of Figure 3 shows the filtered (1–50 Hz) EEG from the C3 electrode for the 0.15–1.0 s after the cue is presented. The second row shows the PSD computed from the raw EEG. The z-scores of the PSD are shown in the third row as inputs to the actor. The first column shows the filtered EEG and processing after an “open” cue. Similarly, the second column shows the filtered EEG and processing after a cue of “close” was shown. The features for the cue of “close” correspond to lower power, in general, than the features of the cue for “open”; in the sample trial of the SCI subject, 44 of the 1 Hz bins have lower power for the “close” cue.

**Figure 5:**
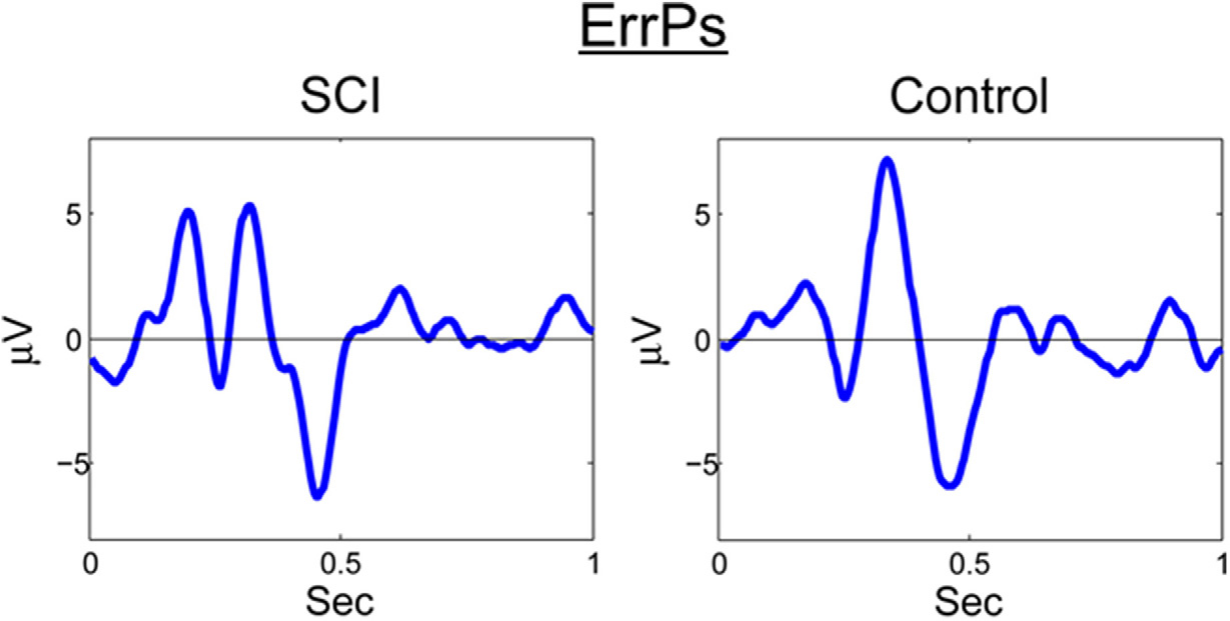
**ErrP for SCI and control**. Averaged across all trials and error-minus-correct for the SCI and control subjects.

**Figure 6:**
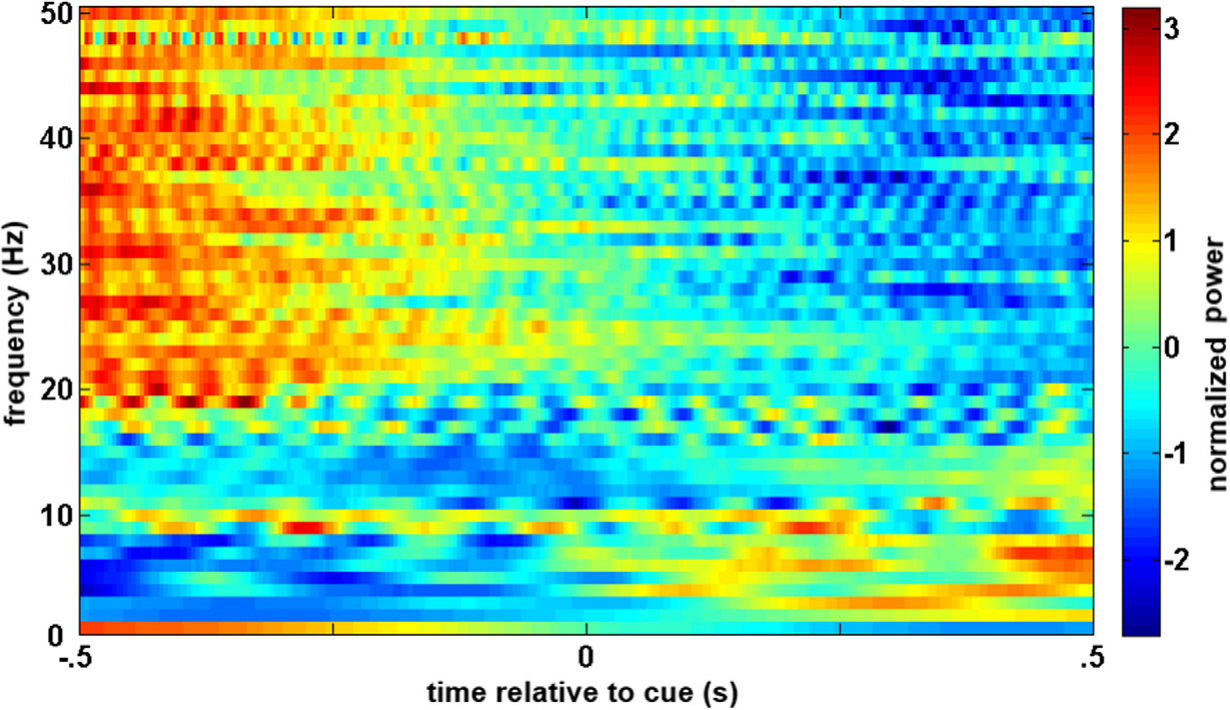
**Averaged trials from closed-loop sessions for cues**. Averaged PSD for cue of “open” for both the SCI and control subjects averaged across all trials.

A similar process was used for inputs to the critic. The first row of Figure 4 shows the filtered, 1–12 Hz, EEG from the Cz electrode for the 0.15–0.70 s after the feedback was shown. PSD of the raw EEG was computed from the Cz electrode, shown in the second row. Finally, the inputs to the critic are shown in the third row as z-scores of the PSD from the Cz electrode. The first column shows the filtered EEG and processing after the feedback of “correct” was presented. The second column shows the filtered EEG and processing for feedback of “error.” Notice that the error potential has a biphasic shape characteristic of this neural oscillation. The features for feedback of “correct” correspond to lower power, in general, compared to features of “error;” in the sample trial for the SCI subject, all 1 Hz bins except 1, 8, 11, and 12 Hz. Figure 5 shows the ErrPs generated by the users, the average of error trials minus the average of correct trials. The ErrPs collected from the users are similar to published results (Ferrez and Millan, 2008). Figure 6 shows the averaged PSD for the cue of “open” for both the SCI and control subjects averaged across all trials. The averaged PSD changes after the cue relative to the averaged PSD before the cue.

### Performance of the system

**Figure 7:**
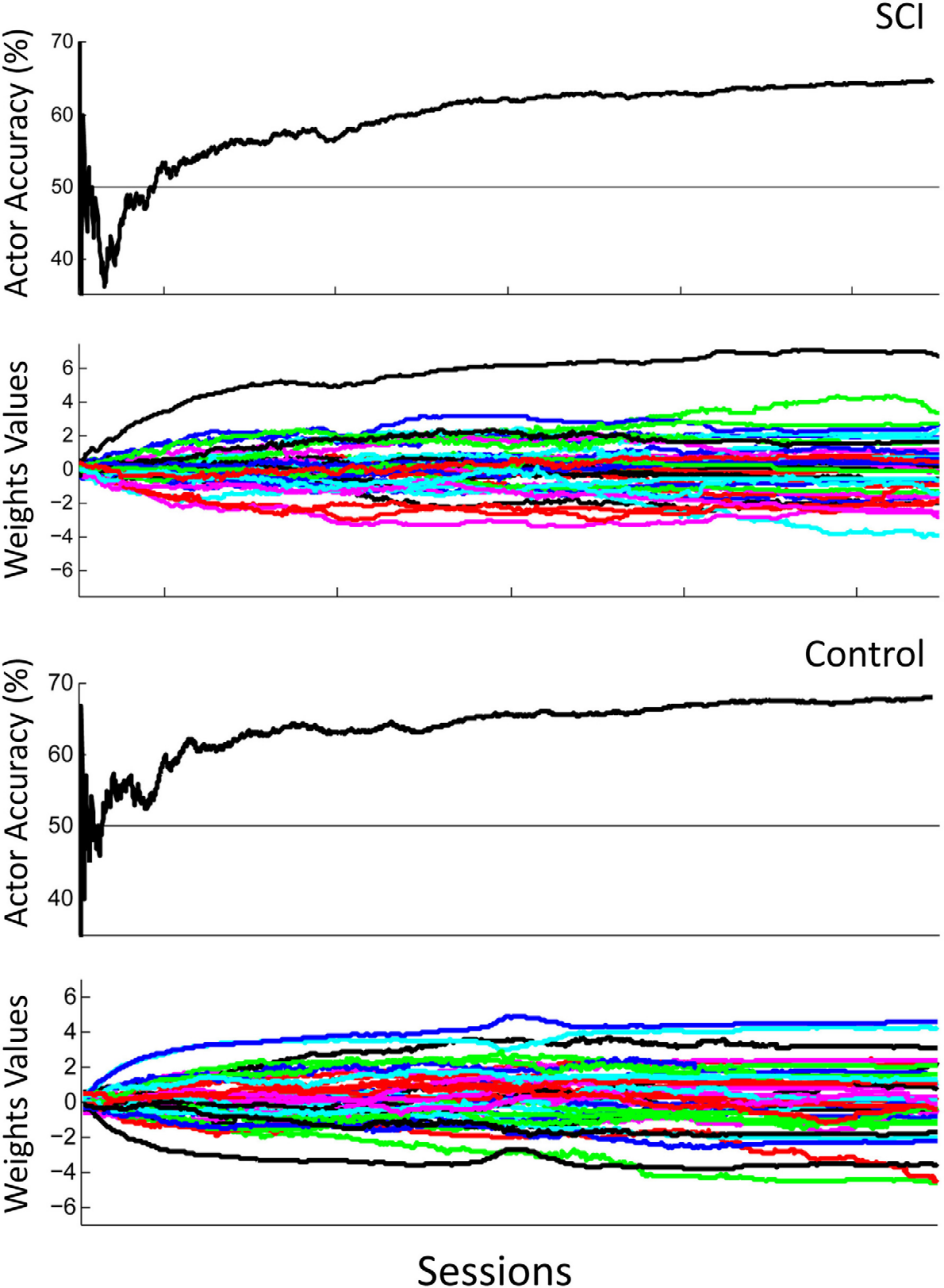
**Actor's performance across 4 sessions**. The first row shows the actor's cumulative classification accuracy and the second row shows the actor's weights adapting for the SCI subject. The third row shows the actor's cumulative classification accuracy and the fourth row shows the actor's weights adapting for the control subject. Final cumulative classification accuracies were significantly above chance (50%) for both subjects (*p* < 0.001, one sided *t*-test).

**Figure 8:**
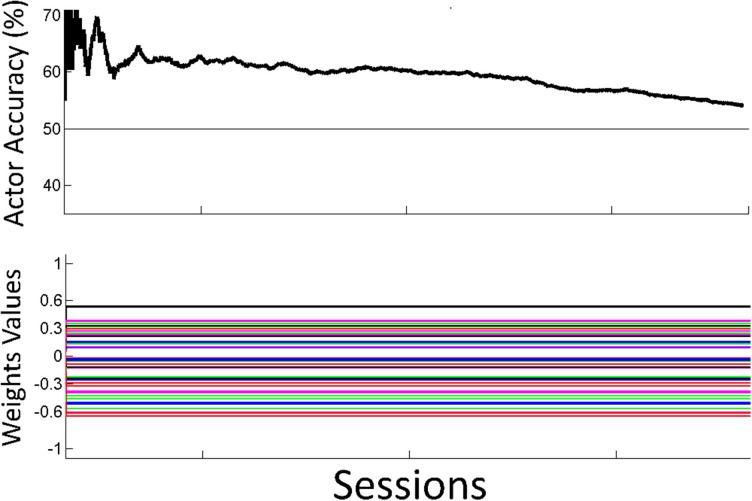
**Off-line analysis with static weights**. Performance for the off-line analysis with static weights trained by supervised learning from data from the preliminary session. Performance was lower than the performance for the adaptive closed-loop system.

Figure 7 shows the overall performance of the actor in classifying motor potentials across 4 sessions, for control and SCI subjects. The classification accuracy starts below 50% (chance level) for the SCI subject, due to the random initial values of the actor's weights. The performance of the actor improves as the actor's weights adapt to feedback from the critic through RL. Over time, the actor's performance approaches the classification accuracy of the critic. Changes in weight values become smaller after the first 2 sessions; however, changes in weight values continue throughout the 1500 trials. The actor made fewer mistakes during the last session than the first, as the actor adapted and learned the user's motor potentials based on feedback from the critic. Figure 8 shows the performance for an off-line analysis with static weights trained by supervised learning from data from the preliminary session. During the first session, the performance was higher for the static weights due to the static weights being trained and the adaptive system not being trained. By later sessions, the static weights have lower performance than the adaptive closed-loop system. The static weights have not been recalibrated while the adaptive system has adjusted its weights.

### Comparison of performance across subjects

**Figure 9:**
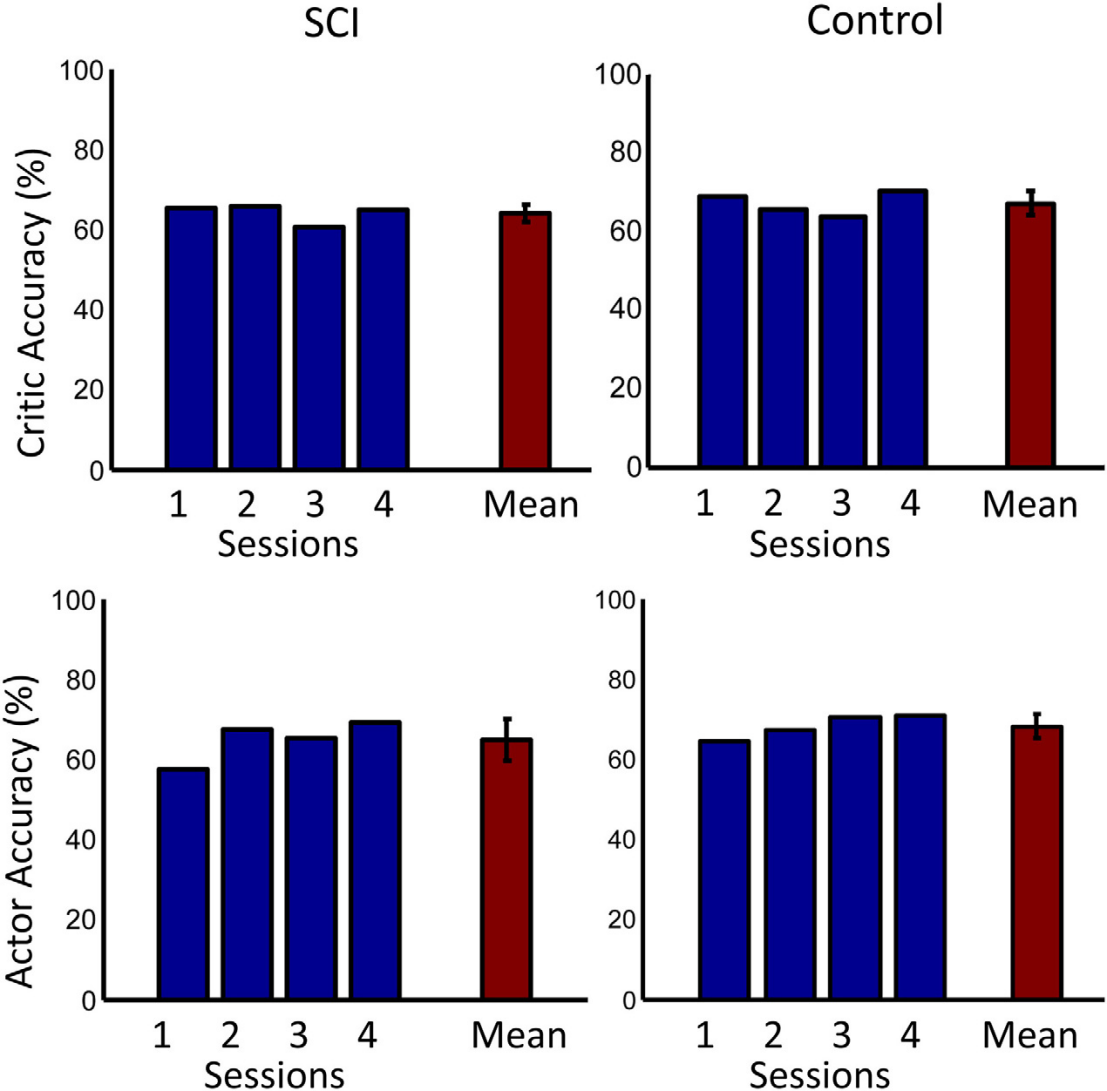
**Accuracies across days**. The first row shows the accuracy of the critic for both the SCI and control subjects. The second row shows the accuracy of the actor. Accuracy for each day is shown in blue. Mean accuracy across days is shown in red. Error bars represent one standard deviation. Mean accuracies were significantly above chance (50%) for both subjects (*p* < 0.001, one sided *t*-test).

The overall performance of both subjects across sessions is shown in Figure 9. Performance was significantly above chance (50%) for both subjects (*p* < 0.001, one sided *t*-test). The performance of the control subject was slightly higher than that of the SCI subject during the first session. This performance difference can be explained by the random initial weight values of the actor more closely matching the desired weight values by chance. The overall performance of the SCI subject was only slightly lower than the control subject, by 0.9%. ErrP classification accuracy was significantly above chance (50%) for both subjects (*p* < 0.001, one sided *t*-test). The system also had lower accuracy for detecting the SCI subject's ErrPs, 64.2%, than for the control subject, 68.8% (*p* < 0.005, one sided *t*-test). This lower performance in detecting the SCI subject's ErrPs could explain the lower overall performance of the SCI subject compared to the control subject. Importantly, the performance of the critic had a small standard deviation, 3.6% for the SCI subject.

## Discussion

This study showed a new EEG based BCI system using RL that was intended for application to control FES and developed as an experimental test bed for augmenting rehabilitation with a BCI. The system used RL to determine the mapping of motor potentials to intended actions based on user generated ErrPs. The BCI continued to adapt to the users throughout the experiment and did not require any offline training after the first session. The ability to adapt to the user without daily initialization could be beneficial in a rehabilitation setting.

After a SCI, the brain experiences measurable maladaptive brain reorganization from disuse (Green et al., 1998; Cramer et al., 2005; Hoffman and Field-Fote, 2006; Kokotilo et al., 2009). These plastic changes can be partially reversed with rehabilitation techniques such as bimanual training and somatosensory stimulation (Hoffman and Field-Fote, 2007, 2010). The motor cortex of chronic SCI subjects also experiences changes when they perform motor imagery training (Cramer et al., 2007). The ability to rehabilitate the motor cortex by motor imagery alone is important in the context of BCI augmented rehabilitation because motor imagery is often used to control BCIs. Notably, motor imagery has been used to control hand grasp FES in BCI systems (Pfurtscheller et al., 2003; Müller-Putz et al., 2005). The combination of motor imagery and BCI controlled FES has been shown to rehabilitate finger extension in a stroke subject (Daly et al., 2009). This improvement occurred with only 3 sessions a week over 3 weeks. Compared to other interventions such as constraint-induced movement therapy, this is a limited amount of time participating in rehabilitation (Liepert et al., 2000). By using an adaptive BCI, the subject could participate in rehabilitation over a longer period of time similar to constraint-induced movement therapy without needing to stop the rehabilitation to recalibrate the system. Other approaches to online adaption have a limited ability to adapt since they rely on supervised learning from a training set (Li and Guan, 2008; Li et al., 2008; Vidaurre et al., 2011). The proof-of-concept presented in this work also opens the possibility for the subjects to take the system home and use it continuously. This is due to not only the continuous RL that does not require calibration by a scientist but also to the design which uses the commercial Bioness H200 and an easy to use wireless Advanced Brain Monitoring EEG system.

In this study, ErrPs were collected from two users and were similar to published results (Figure 5) (Ferrez and Millan, 2008). A classifier to detect the ErrPs during the closed-loop sessions was created (Table 1). Feedback from the ErrP classifier was used to adapt the system to the user using RL (Figure 7). The system was able to classify both single trial ErrPs and motor potentials from features created from EEG recordings (Figures 3, 4). The performance of the system improved over successive sessions until the performance reached the accuracy level of the ErrP classifier (Figure 9, row 2). Maintaining continuity in the performance over time is a critical aspect in the rehabilitation process. The user is able to pick up from the last level of progress achieved from the previous session.

Several additional results are also applicable to the use of the system during rehabilitation. The weights' values during later trials became stable, meaning the user would not experience sudden decreases in performance (Figure 7, row 2). The weights continued to adapt even in later trials, so the system can be expected to continue to adapt to the user in future trials, and during rehabilitation. The performance of the system increased above chance during the first day and continued to show improvement in later trials, both factors in maintaining user motivation and engagement (Figure 7, row 1). In the present architecture, the performance of the motor potential classifier is limited by the performance of the ErrP classifier. However, the ErrP classifier could perform better than the motor potential classifier then the limiting factor would be the motor potential classifier's performance. While this study provides proof of concept, future work is focused on extending the paradigm to additional rehabilitation settings of asynchronous tasks that do not require the use of cues and limiting the update of algorithm weights to when the detection of an ErrP is above a threshold.

### Conflict of interest statement

The authors declare that the research was conducted in the absence of any commercial or financial relationships that could be construed as a potential conflict of interest.
